# COVID-19 vaccine acceptance and associated factors among pregnant and lactating women attending maternity care clinics in refugee camps in Jordan

**DOI:** 10.1371/journal.pone.0305314

**Published:** 2024-06-11

**Authors:** Alaa Dalky, Tamara Osama Quran, Sawsan Abuhammad, Haneen Mahyoub Al-Faraj, Salam Bani Hani, Mohammed ALBashtawy, Imad Rasheed Abu Khader, Mohammed Jallad, Basma Salameh

**Affiliations:** 1 Faculty of Medicine, Department of Health Management and Policy, Jordan University of Science and Technology, Ar-Ramtha, Jordan; 2 Jordan University of Science and Technology, Irbid, Jordan; 3 Faculty of Nursing, Department of Maternal and Child Nursing, Jordan University of Science and Technology, Irbid, Jordan; 4 Faculty of Medicine, Department of Health Management and Policy, Jordan University of Science and Technology, Irbid, Jordan; 5 Faculty of Nursing, Nursing Department, Irbid National University, Irbid, Jordan; 6 Princess Salma Faculty of Nursing, Department of Community and Mental Health, Al al-Bayt University, Mafraq, Jordan; 7 Faculty of Graduate Studies, Arab American University, Ramallah, Palestine; 8 Faculty of Nursing, Arab American University, Jenin, Palestine; Mutah University, JORDAN

## Abstract

**Background:**

Despite the advantages of vaccination in preventing maternal and fetal problems, there were many concerns in the medical community regarding vaccine safety for pregnant women, and this has put obstetricians in a challenging situation when it comes to advising their pregnant patients on whether to obtain the vaccine

**Aim:**

This study was performed to define the level of acceptance of COVID-19 vaccination and assess the impact of COVID-19 attitudes and knowledge on vaccine acceptance between pregnant and lactating Syrian women who are seeking prenatal care services at the clinics in Azraq refugee camp in Jordan.

**Method:**

A quantitative, cross-sectional study utilizing a non-probability convenience sample. A validated and reliable self-administered questionnaire consisting of four sections was used.

**Results:**

A total of 412 pregnant/lactating women was recruited The acceptance rate of the COVID-19 vaccine among participants was 86.5%. There was a significant positive moderate association between respondents’ attitudes and knowledge around the COVID-19 vaccine and their acceptance of the vaccine (r = .468, p < .001, r = .357, p < .001), respectively.

**Conclusion:**

To effectively mitigate the COVID-19 pandemic and achieve collective protection, decision-makers must intensify the efforts in promoting the importance of maternal vaccination, especially in vulnerable communities that suffer the most from pandemic outcomes.

## Introduction

Globally, the coronavirus pandemic is a leading cause of sickness and death [1]. With a case-fatality ratio (CFR) of 0.91%, the global cumulative incidence of COVID-19 as of December 2, 2023, was 772,143,014 reported cases and 7,003,610 associated deaths. In the meantime, 351,822 related deaths (CFR 1.5%) and 23,406,897 cases (representing 3.03% of the worldwide total) have been reported from the Eastern Mediterranean Region (EMR) [2]. Additionally, the COVID-19 pandemic had a major effect on pregnant women’s mortality rates in 2021 [3]. According to data, the number of maternal deaths in the United States increased by 40% in 2021 to over 1,200, up from 861 in 2020 and 754 in 2019. This spike is partly linked to COVID-19 difficulties that made prenatal patients’ preexisting health issues worse [4].

With a population of about 10 million, Jordan has one of the highest per capita rates of COVID-19 infection worldwide. In 2021, there were over 500,000 cases and over 8,000 fatal cases reported [5]. Thus, vaccination is an important technique for limiting infections and reducing illness severity in the COVID-19 epidemic [6]. Early studies revealed that pregnant women with COVID-19 are more likely to experience maternal complications such as antepartum hospitalization, intensive care unit admission, mechanical ventilation, and death than age-matched non-pregnant women, along with obstetric problems such as preterm birth and fetal death [7–10]. So, vaccination against infectious illnesses is a very successful public health approach that has been shown to considerably decrease infection-related worldwide morbidity and mortality [11].

Attitudes and knowledge about COVID-19 vaccination vary significantly across different populations and regions, with several factors influencing perceptions and acceptance rates. for instance, in a study performed by Chisale, Kambalame [12] in Malawi, 95.7% of participants knew about the COVID-19 vaccine, indicating that most participants had a good understanding of vaccination. Although just 38.7% had received the vaccination, roughly 68.4% of respondents thought it was successful. Besides, A community-based study conducted in Yemen by Hassan, Al-Aghbari [13] found differing opinions about the COVID-19 vaccination. Although 92% of participants were willing to adhere to COVID-19 requirements, roughly 61% were reluctant to get the vaccination because of mistrust, rumors, and worries about its safety.

Although there were still substantial obstacles to uptake, healthcare professionals in northeastern Ethiopia had generally positive knowledge and attitudes regarding COVID-19 immunization. Several variables, including sociodemographic traits, knowledge levels, and attitudes toward the vaccine, affected how likely they were to get vaccinated [14].

Despite the advantages of vaccination in preventing maternal and fetal problems, there were many concerns in the medical community regarding vaccine safety for pregnant women, and this has put obstetricians in a challenging situation when it comes to advising their pregnant patients on whether to obtain the vaccine [15]. The acceptance of COVID-19 vaccines among pregnant and lactating women in refugee camps in Jordan is a critical public health issue. This population is particularly vulnerable due to several factors, including limited access to healthcare, lower socioeconomic status, and potential exposure to misinformation. Ensuring high vaccine uptake in this group is essential for protecting both maternal and child health and for controlling the spread of COVID-19 within refugee camps. However, pregnant and lactating women had a greater acceptance rate than the general public, according to studies done in India, China, Qatar, and South American nations [16–18]. In contrast, acceptance rates were low in studies performed in North America, Europe, Russia, and Australia [19]. Therefore, numerous variables influence the degree of uptake of COVID-19 vaccines such as demographical factors of age, sex, and geographic location [14]. Socio-economic status such as income level and employment [12]. Besides, educational attainment and awareness campaigns are effective in public health [20]. By addressing the above-mentioned variables health authorities can improve COVID-19 vaccine uptake among pregnant and lactating women in refugee camps, thereby enhancing maternal and child health outcomes and contributing to the broader effort to control the pandemic. In this study, the associations between COVID-19 vaccine knowledge, attitudes, and acceptance were investigated.

## Material and methods

### Study design

A quantitative, cross-sectional study was done.

### Settings

This study was conducted at two antenatal clinics located in villages # 3 and # 6 of the Azraq refugee camp, which was built for Syrian refugees escaping from the civil conflict in Syria and is located near Azraq, Jordan. Over 41,000 Syrian refugees are living in the Azraq camp in Jordan, and a sizable number of pregnant women arrive there every year. Comprehensive prenatal care is offered at the camp via a range of medical services run by UNHCR and affiliated organizations (WHO, 2022). According to Jordan Azraq’s annual reports, it was reported that 1,647 pregnant women received antenatal care in their first trimester [21].

### Study participants

The participants of this study were pregnant and lactating women aged 18 years and above, attending antenatal clinics at village # 3 and village # 6 of the Azraq refugee camp. Participants were included if they met the following criteria, namely (1) Syrian refugees residing at the Azraq refugee camp, (2) Pregnant or lactating attendees of the antenatal clinics at Village # 3 and Village # 6 aged at least 18 years. On the other hand, any women aged less than 18 years, neither pregnant nor lactating attendees of the clinics were excluded from the study.

### Sample size

The calculation of the sample size for the current research was determined using the following formula [22]: (n) = (Z^2 * SD (1-SD) / e^2). In which (n) is the sample size, Z = z score, and e = confidence interval. Thus, by assuming a 95% confidence level, 0.5 standard deviations, and a margin of error (confidence interval) of +/- 5%, the sample size was calculated as follows:

((1.96)2 x .5(.5)) / (.05)2 = (3.8416 x .25) / .0025 = .9604 / .0025 = 384.16 Patients = (385).

### Recruitment strategies

United Nations High Commissioner for Refugees (UNHCR) operates two Syrian refugee camps in Jordan, Za’atari Camp in Mafraq, hosting over 80,000 refugees, and Azraq Camp near Azraq, hosting approximately 40,000 refugees. According to UNHCR, only 18% of refugees in Jordan live in refugee camps. We used convenience sampling for this study and asked women who met the sampling criteria to fill out the questionnaire. However, after obtaining the needed approvals Institutional Review Board (IRB) of Jordan University of Science and Technology, the Ministry of Health (MOH), the Syrian Refugees Affairs Directorate (SRAD), and UNHCR, SRAD approved collecting data from only two villages of the camp, which are Village # 3 and Village # 6. Data collection was started on 11/10/2022 and ended on 15/04/2023.

### Measurements

A self-administered questionnaire consisted of four main parts employed for primary data gathering. The first portion covers demographic data, including variables such as age, respondent’s condition, pregnancy trimester, postpartum period, mode of delivery, mode of feeding infant, education level, employment status, number of children, chronic disease condition, and flu vaccination. The subsequent parts provided details about the study variables, including the knowledge, attitudes, and acceptance towered the COVID-19 Vaccine, in the second, third, and fourth sections, respectively. To assess the knowledge of COVID-19 vaccination there were five items in the knowledge part, and there were three possible answers: "Yes," "No", and "Don’t know." The response "yes" was scored as 3, and the responses "no" as 2, and "don’t know" as 1. If the mean score is between 1.00 and 1.66 it is ranked as low knowledge, between 1.67 and 2.33it is ranked as moderate knowledge, and if it is between 2.34 and 3, this is scored as high level of knowledge.

The attitudes section in this study consisted of 24 items, all of which were scaled using a Likert scale from 1 to 5. Where 1 = strongly disagree, 2 = disagree, 3 = neutral, 4 = agreed, and 5 = strongly agree. Where the mean from 1 to 2.99 is considered a general negative attitude, and above 3 is a general positive attitude.

For the acceptance of COVID-19 vaccination, this section included three items with three possible answers (yes/no/uncertain), where yes was coded as 3, no as 2, and uncertain as 1. If the mean score was between 1.00–1.66 it is considered a low degree of agreement, between 1.67 and 2.33 it is considered moderate, and between 2.34 and 3 it is considered a high acceptance level.

Two approaches have been used to measure the study tool’s reliability, Cronbach’s alpha was used for Likert scale answers while Kuder-Richardson ver 20 was used for binary scale answers and the result in Table 1 shows that all study tools’ have a reliability coefficient above (α = 0.70) which indicates that the tools have a reasonable reliability index.

**Table 1:** Reliability analysis.

Scales	Knowledge about the COVID-19 vaccine	Attitudes towards the COVID-19 vaccine	COVID-19 vaccine acceptance
**Number of items**	8	24	3
**level of measurement**	Binary	5-points Likert scale	Binary
**Reliability value**	KR = .789	α = .857	KR = .726

KR = Kuder Richardson 20, α = Cronbach’s alpha.

### Ethical considerations

Ethical permission to conduct this study was obtained from the Human Research Ethics Committee (HREC) at Jordan University of Science and Technology in Jordan on 11/09/2022. Therefore, this study has Institutional Review Board (IRB) approval in addition to approvals from the MOH, SRAD, and UNHCR. The researcher communicated with the concerned parties and informed them of the intended study including its significance, purpose, and other relevant details. In this study, participation was entirely voluntary after the completion of a written informed consent. The study’s purpose, target audience, significance, researcher name, and other pertinent information were all included in the first paragraph of the participant questionnaire written by the author. A clear question about participation was also included. The researcher advised the participants that their participation in the study was voluntary and that they could leave at any time to control risk. Additionally, all study participants were given code names to maintain their anonymity and confidentiality. Informed consent was obtained from all the participants.

### Statistical analysis

Statistical Package for Social Science version 25 was used to analyze the collected data. Data was checked for missing and inconsistency. Descriptive statistics of means, frequency, standard deviation, and percentages were used to describe categorical and continuous demographics. Inferential statistics of the Person produces moment correlation to assess the relationship between knowledge and Acceptance and between Attitudes and Acceptance. P-value was significant at < 0.5.

## Results

### Response rate & demographic characteristics of respondents

Out of 450 respondents reached, 412 agreed to participate. While 17 responses were excluded from data analysis, in which (n = 10) respondents under the age of 18, (n = 5) neither pregnant nor lactating, and (n = 2) were Jordanian clinic staff, another (n = 10) responses were excluded because the participant withdrew from the study. A total of 385 with a response rate of 91.6% questionnaires were finally included in the analysis.

The descriptive data of the respondents’ demographics are displayed in Table 2 section 1. All participants were Syrian females, 43.1% were born between 1990 and 1999, and 23.9% were younger than 22 years old. Around 77.7% of participants had a primary/secondary school education level, and 86.2% were unemployed. Of the responders, 35.8% were nursing babies and 64.2% were pregnant. A little over 88.3% are parents, and 93.2% are free of chronic illnesses. Of those who received the COVID-19 vaccine, almost 89.9% received both doses, while 84.9% did not receive the flu shot.

**Table 2:** Descriptive analysis of the study variables (n = 385).

Descriptive analysis of the demographics and characteristics of study participants				
Demographics Items	F (%)			
**Year of Birth** less than 1980 1980–1989 1990–1999 2000 and more	5 (1.30) 122 (31.7) 166 (43.1) 92 (23.9)			
**Choose what applies to your condition** Currently pregnant Currently breastfeeding	247 (64.2) 138 (35.8)			
**Education level** Illiteracy Bachelor’s degree or Higher Associate degree Primary/secondary school	20 (5.20) 43 (11.2) 23 (6.00) 299 (77.7)			
**Employment status** Unemployed Employed	332 (86.2) 53 (13.8)			
**Do you have children?** No Yes	45 (11.7) 340 (88.3)			
**Do you have any chronic diseases?** No Yes	359 (93.2) 26 (6.80)			
**Have you taken the COVID-19 vaccine?** No Yes (First dose) Yes (Both doses)	23 (6.00) 16 (4.20) 346 (89.9)			
**Have you taken the flu vaccine?** No Not sure Yes	327 (84.9) 35 (9.10) 23 (6.00)			
**Descriptive analysis of the COVID-19 vaccine acceptance items**				
**Items Description**	**No**	**Yes**	**Mean**	**SD**
	**F (%)**	**F (%)**		
Q1. Do you accept to take the COVID-19 vaccine?	52 (13.5)	333 (86.5)	2.76	0.630
Q2. Do you accept to get the COVID-19 vaccine while pregnant?	363 (94.3)	22 (5.70)	1.21	0.530
Q3. Do you agree/accept to take the COVID-19 vaccine while lactating?	157 (40.8)	228 (59.2)	2.41	0.779
Total COVID-19 vaccine acceptance mean score			4.51	0.780
**Descriptive Analysis of Knowledge about the COVID-19 vaccine**				
**Items Description**	**No**		**Yes**	
	**F (%)**		**F (%)**	
**Q1.** It is essential to take the COVID-19 vaccine.	101 (26.2)		284 (73.8)	
**Q2.** Do you think that the COVID-19 vaccine brings the probability of risk to your baby?	235 (61.0)^a^		150 (39.0)	
Q3. The COVID-19 vaccine protects against infectious diseases.	219 (56.9)		166 (43.1)	
Q4. Does the COVID-19 vaccine produce long-term term immunity?	232 (60.3)		153 (39.7)	
Q5. Does the COVID-19 vaccine increase allergic reactions?	313 (81.3)		72 (18.7)	
Q6. Does the COVID-19 vaccine increase autoimmune diseases?	328 (85.2)^a^		57 (14.8)	
Q7. Is it dangerous to use an overdose of the COVID-19 vaccine?	255(66.2)		130(33.8)^a^	
Q8. Post-vaccination there is no need to follow preventive measures (wearing masks and social distancing)	190(49.4)^a^		195(50.6)	
**Total COVID-19 vaccine Knowledge mean score**	(M = 4.69, SD = 1.57)			
**Descriptive analysis of the Attitudes towards the COVID-19 vaccine items**				
**Items**	M		SD	
Q1. A coronavirus vaccination must be obligatory for all pregnant and lactating women	2.42		1.319	
Q2. Without being vaccinated against COVID-19, I would possibly have acquired COVID-19.	2.85		1.070	
Q3. If I am vaccinated against COVID-19, my child/fetus and I will be protected from COVID-19.	3.06		1.185	
Q4. If I don’t get vaccinated against COVID-19 resulting in COVID-19 for me and my kid/fetus, I will remorse not getting vaccinated.	3.97		1.117	
Q5. It would be very easy for me to get vaccinated against COVID-19.	4.61		0.791	
Vaccination against COVID-19 may infect me and my child/fetus with COVID-19.	2.65		1.099	
I will be concerned about suffering from the side effects of the COVID-19 vaccination for my child/fetus.	4.17		1.012	
I may regret getting a vaccine against COVID-19 if I, my kid/fetus later suffer from side effects from the vaccination.	4.28		1.030	
COVID-19 vaccination would be too novel for me to be assured about getting vaccinated	3.77		1.106	
Most people will permit pregnant and breastfeeding women to receive the vaccine against COVID-19	2.59		1.259	
Other people like me will not permit pregnant and breastfeeding women to acquire a vaccine against COVID-19	3.41		1.324	
In general, vaccination is a good thing	3.83		.978	
I’m afraid of needles	3.16		1.527	
If I get vaccinated, I don’t think I will need to follow social distancing and other constraints forced by COVID-19	3.06		1.422	
I know sufficient about COVID-19 to make an informed decision about whether to get vaccinated	4.04		1.003	
I know enough about the COVID-19 vaccine to make an informed decision about whether to get vaccinated.	3.99		1.051	
Only pregnant and breastfeeding women who are at risk of serious disease from COVID-19 need to be vaccinated	2.68		1.060	
My family will accept to get me vaccinated against COVID-19.	4.03		1.065	
My friends will agree to get me vaccinated against COVID-19.	4.07		0.980	
If the government endorses the COVID-19 vaccination for pregnant and lactating women, I will get vaccinated	3.48		1.192	
If a healthcare professional recommends a COVID-19 vaccination for mothers like me, I’ll get vaccinated	3.85		1.139	
The COVID-19 vaccination is just a method to make money for vaccine producers.	2.72		1.118	
The COVID-19 vaccine will permit us to return to normal life	4.01		.956	
There will be no point in getting vaccinated against COVID-19 unless I can return to my normal life	3.78		1.072	
**Total attitude towards the COVID-19 vaccine mean score**	**3.19**		**0.55**	
**Descriptive analysis of specific questions for pregnant and lactating respondents**				
**Gestational age for pregnant respondents (n = 247)** 1^st^ trimester 2^nd^ trimester 3^rd^ trimester	F (%) 65 (16.9) 99 (25.7) 83 (21.6)			
**Postpartum period for lactating respondents** **(n = 138**) Less than 6 weeks More than 6 weeks	37 (9.60) 101 (26.2)			
**Mode of delivery for lactating respondents (n = 138)** Normal vaginal delivery Cesarean delivery	88 (22.9) 50 (13.0)			
**Mode for feeding infant for lactating respondents (n = 138)** Breast milk feeding Formula feeding Supplementary feeding Any Combination	112 (29.1) 3 (0.80) 2 (0.50) 21 (5.50)			

### Descriptive analysis of the COVID-19 vaccine acceptance

Respondents have unfavorable attitudes toward question #2 since its mean is lower than the mean of the scale (2). On the other hand, respondents have favorable attitudes toward the remaining questions since their respective means are higher than the mean of the scale (2). The results have shown that 86.5% of the sample has accepted to take the COVID-19 vaccine, however, the acceptance level was higher between lactating than pregnant women (5.7% vs.59.2%), nevertheless, the overall scale mean score was (M = 4.51±0.78) revealing that their acceptance was in above moderate level Table 2.

### Descriptive analysis of the knowledge about the COVID-19 vaccine

In terms of Syrian women refugees toward COVID-19 vaccine knowledge, the findings in Table 2 Section 3 have shown that (61.0%) reported that the vaccine does not have any harm to babies, (>80.0%) mentioned it does not cause allergic reactions or autoimmune diseases while low percentage of sample reported correctly that the vaccine protects against infectious diseases 43.1%, produce long term-immunity 39.7%, and it is dangerous to use an overdose 33.8%. However, fifty-fifty exhibited that there is no need to follow preventive measures after taking the shot, generally, the mean knowledge score found to be (4.69 out of 8) with only 58.4% of corrected answers have been observed.

### Descriptive analysis of the attitudes towards the COVID-19 vaccine

Descriptive statistics of all the attitudes regarding the COVID-19 vaccine items are shown in Table 2, section 4. The data show that respondents have negative attitudes toward questions # 1, # 2, # 6, # 10, # 17, and # 22) because their respective means are lower than the scale mean (3). On the other hand, respondents have favorable attitudes toward the remaining questions since their respective means are higher than the mean of the scale (3). The highest mean score was related to the item “It would be very easy for me to get vaccinated against COVID-19” with an average of (M = 4.61±0.791), while the lowest score was related to the item related to “A coronavirus vaccination must be obligatory for all pregnant and lactating women” with an average of (M = 2.42± 1.319).

### The relationship between knowledge and COVID-19 vaccine acceptance among pregnant and lactating Syrian women

Pearson product-moment correlation was applied to explore the correlation between knowledge and COVID-19 vaccine acceptance since the data was normally distributed. The results have shown there is a significant moderate positive correlation (r = .357, p < .001, n = 385) as the person’s knowledge score increases, the COVID-19 vaccine acceptance increases as well. Additionally, the results revealed that there is a significant moderate positive association between attitude and COVID-19 vaccine acceptance (r = .468, p < .001, n = 385) revealing as their attitude increases, their acceptance of the vaccine will increase as well Table 3.

**Table 3:** Parson correlation test (knowledge and acceptance; attitudes and acceptance).

**Knowledge of COVID-19 vaccine**		**COVID-19 vaccine acceptance**
r		.358
p-values		< .001
N		385
**Attitude toward COVID-19 vaccine**	**COVID-19 vaccine acceptance**	
r	.468	
p-values	< .001	
N	385	

if r < 0.30, the correlation is considered weak, 0.30–0.70 is moderate, > 0.70, is a strong correlation.

## Discussion

Based on our findings, the COVID-19 vaccine acceptance level was 86.5%, these results were reinforced by a similar recent study conducted in the Zataari refugee camp in Jordan, which documented that the COVID-19 vaccine acceptance among Syrian refugees was 89.6%, which is higher than the local Jordanian population [23]. Moreover, a cross-sectional study done in Eastern Ethiopia at the beginning of 2021 also detected that the acceptance rate was 62.2% [24]. Besides, a similar study was performed to define the level of acceptance of COVID-19 vaccination and discover the elements that impact vaccine acceptance among pregnant women in Saudi Arabia; among around 5000 participants, the vaccine acceptance rate was 68% [25]. However, another study performed by Takahashi, Ishitsuka [26] observed vaccine hesitancy in 51.1% of pregnant women. Also, pregnant respondents to a study done in 2021 by Sutton, D’Alton [27] had the lowest proportion of vaccine acceptance (44.3%; P<0.001), and lactating participants were the second most likelihood of receiving the vaccination (55.2%).

In this study, the average score of pregnant women toward vaccination was (M = 4.69, SD = 1.57), revealing poor knowledge. This result was consistent with a study conducted among seven low and middle-income countries that found poor knowledge about the effectiveness and safety of vaccination [28].

By explaining and discussing the results of our current study in light of previous studies, it was found that few of the previous studies were incompatible with our results [29]. However, it is essential to take into consideration many factors that might contribute to these findings. For instance, a study was done almost a year before, and in the COVID-19 pandemic, timing is critical. As time passes, more is known about both the virus and the vaccines, which may contribute to higher vaccine acceptance. Also, more vaccine safety data is published, which comforts the population and encourages them to receive the vaccine. Thus, it is essential to note that these factors might contribute to the variation in recent studies’ findings in addition to the respondents’ demographics and study setting [26].

The authors also discuss the efforts made by official parties in the study setting to raise the community’s awareness of the COVID-19 vaccine and its significance in ending the epidemic. In such crowded places, efforts need to be condensed to stop the spread of infections as soon as possible. Hence, the UNHCR, along with the Jordanian government and other partners, directed their measures toward increasing COVID-19 vaccine acceptance and uptake [21]. Also, in my opinion, the Jordanian defense law and the rules compelled by the UNHCR and SRAD in refugee camps to stop the spread of the pandemic played a vital part in increasing the COVID-19 vaccine’s acceptance and uptake. As most of the official paperwork for refugees, like work permits and volunteering opportunities, required the applicant to have a vaccination certificate, this led to higher vaccine uptake rates. This being said vaccine acceptance has an essential role in ending the COVID-19 pandemic and reaching herd immunity. Especially in vulnerable communities that face more alarming outcomes of the pandemic.

Our study detected a relationship between knowledge and COVID-19 vaccine acceptance among pregnant and lactating Syrian women. Pregnant women may be more receptive to vaccination against COVID-19 if they have access to trustworthy information from trained healthcare professionals on the existing level of knowledge about safety, efficacy, and the recommendations of scientific sources. Rather than emphasizing the danger of the illness when prescribing a vaccination, it may be more effective for community health campaigns to emphasize the preventive function and protection of the vaccine between pregnant and nursing women [30]. This finding is supported by another research was conducted in 2022, they found that respondents with a high level of knowledge regarding COVID-19 inclined to vaccinate more than respondents with a low level of knowledge. But their study was done in another refugee camp, included both sexes and wasn’t targeted to pregnant and lactating women only [23]. Furthermore, numerous studies have shown that in the general population, higher levels of education correlate with increased acceptance of COVID-19 vaccinations [31,32]. Others suggested that adequate knowledge is essential for the formation of preventative knowledge and positive attitudes and actions, while a lack of knowledge increases the risk of infection. But fewer studies in the literature had a full sample of pregnant and lactating refugee women, and our finding may be due to the low socio-economic level of this vulnerable population [33,34].

On the other hand, this finding is incompatible with research done in Western China, which indicates that COVID-19 immunization acceptability is greater among pregnant women with lower levels of education. While pregnant women with a higher degree of education exhibited more vaccination refusal, which was correlated with their awareness of the COVID-19 vaccine, they may have encountered a greater amount of adverse information related to the COVID-19 vaccination, given the fact that 77.7% of our study participants had a primary or secondary school education level [35]. Also, it was found that although their study’s respondents had inadequate knowledge towered specific facts about the COVID-19 vaccine, most of them approved that it was important to be vaccinated for COVID-19 and were conscious that pregnant and breastfeeding women are eligible for vaccination [17].

Our study found a relationship between attitude and COVID-19 vaccine acceptance among pregnant and lactating Syrian women, this finding is supported by the studies done in Jordan, which found a positive attitude among participants toward the COVID-19 vaccine [23,36]. Besides, a study done in Malta discovered a strong association among participants’ beliefs that vaccination will aid save people against COVID-19 and their readiness to obtain the vaccine, as well as a significant association between preparedness to have the vaccine and giving importance to the thoughts of family and friends and with the significance given to the guidance of health professionals [37]. Other studies found similar findings in southwest Ethiopia, where the attitudes of pregnant women toward the COVID-19 vaccine were assessed. The findings of this study revealed that more than half, 66.7%, of pregnant women had a positive attitude toward the vaccine. In Egypt, 74.9% of research participants had a positive attitude; nonetheless, this percentage was greater than in studies done in communities in southern Ethiopia (54.5%) and northern Ethiopia (57.5%) [38]. This may be attributed to the fact that expecting women may strictly adhere to the counseling/advice of healthcare professionals during prenatal care visits on COVID-19 preventative measures [38–41]. In the last, it was shown that pregnant women who had a positive attitude towards the COVID-19 vaccine were 2.1 times more likely (AOR: 2.128, 95% CI: 1.348, 3.360) to express an intention to get the COVID-19 vaccination, as opposed to those with a negative attitude [42].

## Conclusion

The results of the study indicate that immigrant women’s acceptance and their awareness of the COVID-19 vaccine are very positively correlated. Furthermore, a positive association was discovered between women’s perceptions of the COVID-19 vaccine and their level of acceptance. Therefore, to put a stop to the pandemic and establish herd immunity, decision-makers must concentrate their efforts on increasing public knowledge and awareness of the COVID-19 vaccine. This holds particular significance in susceptible groups that are most impacted by the pandemic’s consequences.

## Supporting information

S1 Dataset (XLSX)
